# Transcriptomic Analysis of Hemolymph in Pollen-Foraging and Non-Pollen-Foraging *Apis mellifera ligustica* and Identification of Candidate Genes

**DOI:** 10.3390/ani16182909

**Published:** 2026-09-16

**Authors:** Chang Song, Lina Guo, Yuan Guo

**Affiliations:** 1 College of Animal Science, Shanxi Agricultural University, Taigu 030801, China; 2 Shanxi Key Laboratory of Animal Nutrition and Feed Development, College of Animal Science, Shanxi Agricultural University, Jinzhong 030801, China; 3 College of Horticulture, Shanxi Agricultural University, Taigu 030801, China

**Keywords:** *Apis mellifera ligustica*, hemolymph, transcriptome, foraging state, immune-related candidate genes, RNA-seq

## Abstract

Foraging is a physiologically demanding behavior in honey bees, requiring substantial energy expenditure and continuous physiological adaptation. To investigate the molecular responses of hemolymph during foraging, pollen-foraging workers returning from pear flowers and non-foraging workers of *Apis mellifera ligustica* were subjected to hemolymph transcriptome sequencing using the Illumina platform. Differentially expressed genes (DEGs) were identified, followed by Gene Ontology (GO), Kyoto Encyclopedia of Genes and Genomes (KEGG) enrichment analyses, and quantitative real-time PCR (qRT-PCR) validation. A total of 563 DEGs were identified between the two groups, including 323 upregulated and 240 downregulated genes. GO analysis indicated that these genes were mainly associated with proteolysis, peptidase activity, serine hydrolase activity, and cell–cell adhesion. KEGG enrichment analysis showed significant enrichment in lysosome, efferocytosis, protein processing in the endoplasmic reticulum, and metabolic pathways. Unlike classical immune pathways, Toll and Imd signaling showed no significant enrichment. Integrating transcriptomic analysis and qRT-PCR validation identified four candidate genes, *ADAM10-X1*, *Drpr-X2*, *CPT1A*, and *Lipase3*, which are associated with cellular homeostasis, lipid metabolism, and nutrient utilization. These findings demonstrate that foraging primarily induces metabolic and homeostatic remodeling of the hemolymph rather than broad activation of canonical immune pathways. This study provides new insights into the molecular mechanisms underlying physiological adaptation to foraging in *Apis mellifera ligustica* and identifies candidate genes for future studies of honey bee nutritional physiology and behavioral regulation.

## Simple Summary

Honey bees are critical pollinators for farm crops and wild plants. Flying out to collect pollen costs bees large amounts of energy, and this hard work may change their internal physiological state. In this study, we compared circulating body fluid (hemolymph) from worker bees that returned with pollen and bees that came back without pollen. We used gene-sequencing technology to check gene activity differences between these two groups of bees. We found that pollen-collecting bees mainly showed changes in genes linked to energy use and cell maintenance, rather than strong activation of typical infection-fighting immune genes. Four key candidate genes related to fat metabolism and cell cleaning were identified and confirmed. Our work shows that gathering pollen reshapes bees’ internal metabolic status. These results offer new clues for understanding how bees adjust their bodies for field foraging work.

## 1. Introduction

The *Apis mellifera ligustica* (*A. m. ligustica*) is one of the most important pollinators in agricultural ecosystems, and its population stability is directly associated with food security and the maintenance of biodiversity [1,2,3]. However, continuous colony decline worldwide over the past two decades has become a major threat to sustainable agricultural development [4,5]. Extensive studies have demonstrated that this decline is not caused by a single factor but results from the interaction of multiple stressors, including parasitic mites such as *Varroa destructor*, pathogenic microorganisms such as *Nosema ceranae* and deformed wing virus (DWV), pesticide exposure, and nutritional deficiency [6,7]. These pathogens can affect honey bees at different biological levels, ranging from intestinal infection and epithelial damage caused by *Nosema* spp. to systemic and neurological disturbances associated with viral infections, thereby imposing substantial physiological and energetic costs on individual bees and colonies. Within this complex stress network, nutritional immunity is gradually emerging as a key perspective for understanding the mechanisms underlying honey bee health. Nutritional immunity refers to the regulation and allocation of essential nutrients and micronutrients by the host to support immune defense while limiting their availability to invading pathogens. Although insects lack the conventional adaptive immune system found in vertebrates, they possess sophisticated innate immune defenses and can exhibit immune priming, whereby previous exposure to a pathogen or microbial component can enhance subsequent immune responses. The synthesis and maintenance of innate immune effector molecules, such as antimicrobial peptides and phenoloxidase require substantial energy and protein resources [8,9,10], indicating that hosts must achieve precise resource allocation between immune defense and other physiological demands. Therefore, the availability and quality of nutrients directly determine the immune potential of honey bees in responding to environmental stress [11].

Recent studies have further shown that pollen composition can influence fat body morphology, energy substrate levels, antioxidant enzyme activity, and proteolytic processes in honey bees, highlighting the close relationship between pollen nutrition and systemic metabolic and physiological regulation [9,10,11,12,13,14]. However, at the physiological level, hemolymph serves as an important link between nutrient metabolism and immune defense. Honey bees possess an open circulatory system in which hemolymph circulates through the hemocoel and bathes internal organs. Many proteins and other physiological factors are synthesized in tissues such as the fat body and subsequently released into the hemolymph, which transports nutrients, metabolic products, and immune-related factors throughout the body. Thus, hemolymph contributes to metabolism, homeostasis, and immune defense [15]. Specifically, hemolymph contains circulating hemocytes that participate in cellular immune responses, including phagocytosis, nodule formation, and encapsulation [16,17]. Meanwhile, most humoral immune effector molecules—including pattern recognition receptors (PRRs), serine protease cascade components, and terminal effector molecules such as antimicrobial peptides (AMPs) and phenoloxidase (PO)—are synthesized in the fat body and subsequently secreted into the hemolymph cavity, where they exert immune functions [18,19]. Therefore, the hemolymph microenvironment directly reflects the immune defense status of the organism.

More importantly, hemolymph represents the first line of defense against wound infection and invasion of the hemocoel in honey bees [16]. When pathogens penetrate the body wall or peritrophic membrane and enter the hemocoel, pattern recognition receptors (such as PGRPs and GNBPs) in the hemolymph rapidly recognize pathogen-associated molecular patterns (PAMPs), thereby activating serine protease cascade reactions [18,20]. This cascade reaction not only activates prophenoloxidase (ProPO) through proteolytic cleavage to initiate melanization, thereby sealing wounds and eliminating pathogens [21], but also allows some serine protease cascade components to activate Spätzle, further initiating the Toll signaling pathway and inducing the transcriptional synthesis of antimicrobial peptides [18]. In addition, transport proteins in hemolymph (such as transferrin and lipophorin) restrict nutrient acquisition by intracellular pathogens by chelating free iron ions or regulating lipid availability in the environment, thereby constituting an important component of nutritional immunity [22]. Therefore, hemolymph is not merely a carrier of immune molecules; its biochemical composition (such as total protein content, enzyme activity, and metabolite profiles) itself represents a key indicator for evaluating honey bee health status.

However, current studies investigating how natural foraging-related conditions influence hemolymph gene expression remain limited. Previous studies have shown that flight activity in honey bees is associated with changes in gene expression and oxidative damage, while pollen foraging represents an important nutritional input that can influence physiological and metabolic status [23,24]. However, whether these physiological differences are accompanied by systematic changes in the hemolymph transcriptome remains largely unexplored. Therefore, in this study, *A. m. ligustica* were selected as the research subject, and returning worker bees carrying pollen loads (LH group) and returning worker bees without visible pollen loads (CK group) were collected at the hive entrance during the same period. Hemolymph transcriptome sequencing was performed to characterize transcriptional differences between these two sampled groups. This study aimed to identify differentially expressed genes and associated biological pathways between pollen-carrying and non-pollen-carrying returning workers and to identify candidate genes potentially involved in nutrient metabolism and cellular homeostasis. The results provide transcriptomic evidence for understanding molecular differences associated with pollen-carrying status under natural field conditions.

## 2. Materials and Methods

### 2.1. Experimental Insects

Worker honey bees (*A. m. ligustica*) were collected from an ecological pear orchard located in Hongzhiyi Town, Yanhu District, Yuncheng City, Shanxi Province, China (110.99827° E, 35.0151° N) between 28 March and 11 April 2025. The orchard covered approximately 54 hectares and was primarily planted with pear trees. A 0.2-hectare rapeseed field was located approximately 1000 m from the orchard, resulting in a competing floral resource proportion of approximately 0.4%.

The colonies were maintained in six standard Langstroth hives, each containing approximately 3000–3500 adult worker bees. Colony strength was visually estimated based on the approximate number of adult worker bees, and colonies with broadly comparable adult worker populations were selected for the experiment, following commonly used visual approaches for estimating honey bee colony strength. Each colony contained one queen, brood, and available stores of pollen and nectar. The colonies were transported to the pear orchard when approximately 25% of the pear blossoms had opened.

During the peak nectar secretion period (10:00–14:00 each day), returning worker bees were observed at the hive entrance, and the presence, color, and morphology of pollen loads carried in the corbiculae of the hind legs were recorded. Worker bees carrying a visible single type of pollen load were assigned to the pollen-foraging group (LH). Individuals carrying only white or pale green pollen, which was predominant during the pear flowering period, were collected as the pollen-foraging group. Returning worker bees observed at the hive entrance during the same period but without visible pollen loads on their hind legs were assigned to the non-pollen-foraging group (CK). Bees from both groups were collected simultaneously in batches to minimize the influence of environmental variation. Individual bee age and specific foraging task were not determined during field collection.

Immediately after collection, live worker bees were anesthetized on ice at 4 °C for 3–5 min to reduce metabolic activity and delay hemolymph coagulation. A sterile 10 μL micropipette tip was used to puncture the intersegmental membrane at the lateral margin between the second and third abdominal tergites. Gentle pressure was applied to the abdomen to allow hemolymph to exude naturally, after which the hemolymph was immediately collected. No anticoagulant was used in order to avoid potential interference of exogenous chemical reagents with endogenous ribonucleic acid in the hemolymph and subsequent transcriptome sequencing. To preserve ribonucleic acid integrity, all procedures were performed on ice, and hemolymph collection from each individual bee was completed within 2–3 min [25]. Approximately 200 worker bees were collected for each group (LH and CK) during the sampling period. The collected bees were subsequently used to prepare three biological replicates for RNA-seq analysis for each group. Each biological replicate contained 2 g of pooled bee hemolymph samples. All collected hemolymph samples were immediately stored on dry ice for subsequent sequencing experiments.

### 2.2. Sample Quality Assessment and Total Ribonucleic Acid Extraction

Immediately after collection, hemolymph samples were homogenized in TRIzol reagent and stored at −80 °C until use. Total ribonucleic acid was extracted according to the manufacturer’s instructions for the TRIzol reagent. The purity and concentration of total ribonucleic acid were determined using a NanoDrop 2000 spectrophotometer (Thermo Scientific, Wilmington, NC, USA), and ribonucleic acid integrity was evaluated using an Agilent 2100 Bioanalyzer system (Agilent Technologies, Santa Clara, CA, USA). Only samples with a ribonucleic acid integrity number of at least 7.0 were used for subsequent complementary deoxyribonucleic acid library construction.

### 2.3. Sequence Quality Control and Data Filtering

Raw sequencing reads were processed using fastp (version 0.23.4). Reads containing adaptor sequences, reads with more than 10% ambiguous nucleotides, and reads shorter than 50 base pairs after trimming were removed. Low-quality bases with a Phred quality score of 20 or lower at both read ends were trimmed. The resulting high-quality clean reads were retained for downstream analyses. The percentages of bases with Phred quality scores of at least 20 (Q20) and at least 30 (Q30) were calculated to assess sequencing quality, together with guanine–cytosine content.

### 2.4. Sequence Alignment and Transcript Assembly

The reference genome of Apis mellifera (Amel_HAv3.1) and the corresponding genome annotation files were downloaded from the National Center for Biotechnology Information (NCBI) database. A reference genome index was constructed using HISAT2 (version 2.2.1), and clean reads were aligned to the reference genome using splice-aware alignment. Transcript assembly and novel transcript prediction were performed using StringTie (version 2.2.3), which optimizes transcript structures based on a network flow algorithm.

### 2.5. Functional Enrichment Analysis

To investigate the biological functions of differentially expressed genes, Gene Ontology enrichment analysis and KEGG pathway enrichment analysis were performed using the clusterProfiler package (version 4.8.1) in the R environment. Significantly enriched Gene Ontology terms and KEGG pathways were identified using the hypergeometric test, with an adjusted *p* value of less than 0.05 considered statistically significant. In addition, gene set enrichment analysis was performed without applying a predefined differential expression threshold to evaluate the overall enrichment patterns of predefined gene sets between the experimental groups.

### 2.6. Differential Gene Expression Analysis

All samples included biological replicates. Differential gene expression analysis was performed using the DESeq2 package (version 1.42.0) in the R environment. Raw gene count data were normalized using a negative binomial distribution model to account for differences in sequencing depth and library composition. Differential expression between the pollen-foraging and non-pollen-foraging groups was evaluated using the Wald test. Genes with a *p* value of less than 0.05 and an absolute log_2_ fold change of at least 1 were defined as differentially expressed genes and were subjected to subsequent functional enrichment analyses.

### 2.7. Screening and Sequence Analysis of Candidate Genes

Candidate genes were identified by first selecting significantly enriched pathways related to immune function from the KEGG database with an adjusted *p* value of less than 0.05. Differentially expressed genes within these pathways were subsequently screened using the criteria of a *p* value of less than 0.05 and an absolute log_2_ fold change of at least 1. Candidate gene sequences were queried against the NCBI database using the Basic Local Alignment Search Tool to identify the most homologous species together with the corresponding gene names and accession numbers. Open reading frames were predicted using the online Open Reading Frame Finder provided by the NCBI to obtain complete coding sequences.

### 2.8. Quantitative Real-Time PCR Validation

To verify RNA-seq reliability, four differentially expressed candidate genes and one reference gene (Arp1, GenBank: NM_001185145.1) were selected for qRT-PCR. Equal quantities of total RNA were reverse-transcribed into first-strand cDNA with the PrimeScript™ RT Reagent Kit with gDNA Eraser (TaKaRa, Shiga, Japan). Gene-specific primers were designed and synthesized by Shanghai Sangon Biotechnology Co., Ltd. (Shanghai, China) (sequences in Table 1).

The 10 μL qRT-PCR mixture contained 5 μL SYBR^®^ Premix Ex Taq™ II (TaKaRa, Shiga, Japan), 0.5 μL of each forward/reverse primer (10 μM), 1 μL cDNA template, and 3 μL ddH_2_O. Amplification was run on a CFX96™ Real-Time PCR Detection System (Bio-Rad, Hercules, CA, USA): 95 °C for 30 s; 35 cycles of 95 °C for 5 s and 60 °C for 30 s; melting curve analysis (65–95 °C, 0.5 °C/s). Three technical replicates were performed per sample.

## 3. Results

### 3.1. Quality Assessment of Transcriptome Sequencing Data

To characterize transcriptomic differences between pollen-foraging and non-pollen-foraging returning workers of *A. m. ligustica*, high-throughput ribonucleic acid sequencing was performed using the Illumina sequencing platform on six hemolymph samples, including three biological replicates from pollen-foraging workers returning with pear pollen and three biological replicates from returning workers without visible pollen loads. Following stringent quality control, high-quality clean reads were obtained from all samples. The raw and clean base numbers shown in Table 2 were derived directly from the corresponding sequencing reads generated during the sequencing and quality-control procedures. The sequencing output for each sample ranged from 7.07 to 8.67 gigabases. Sequencing quality was consistently high across all samples, with the percentage of bases having a Phred quality score of at least 30 exceeding 97.91% (97.91–98.34%). The guanine–cytosine content ranged from 35.57% to 37.95%, indicating high sequencing quality and reliable data suitable for subsequent transcriptomic analyses.

**Figure 1:**
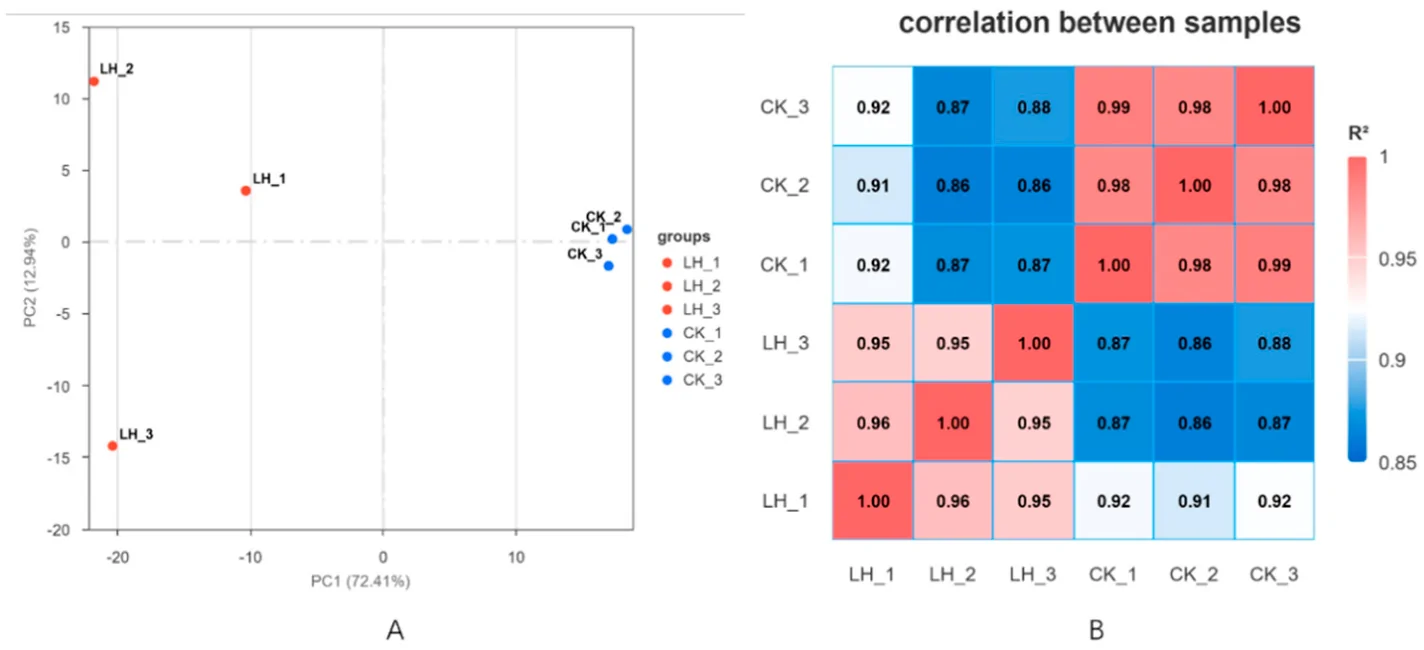
Principal component analysis and Pearson correlation analysis of hemolymph transcriptome samples from *A. m. ligustica* between pollen-foraging and non-pollen-foraging groups. (**A**) Principal component analysis based on normalized gene expression levels. (**B**) Heatmap of Pearson correlation coefficients among all samples. Color intensity represents the strength of the correlation between samples, and the values within each square indicate the corresponding Pearson correlation coefficients.

To further evaluate the consistency among biological replicates and the overall distribution of the samples, Pearson correlation analysis and principal component analysis were performed based on the normalized gene expression matrix. As shown in Figure 1, the correlation coefficients among biological replicates within the pear pollen-foraging group ranged from 0.95 to 0.96, whereas those within the non-foraging control group ranged from 0.98 to 0.99, indicating a high degree of reproducibility and demonstrating the excellent quality of the ribonucleic acid sequencing data. In contrast, the correlation coefficients between the two groups ranged from 0.86 to 0.92, which were noticeably lower than those observed within each group, indicating distinct differences in hemolymph transcriptome expression profiles between the two sampled groups.

Principal component analysis further confirmed the overall separation between the two groups. The first principal component and the second principal component explained 72.41% and 12.94% of the total variance, respectively, accounting for a cumulative 85.35% of the overall variation. Samples from the pear pollen-foraging group and the non-pollen-foraging group were separated along the first principal component, indicating distinct transcriptomic profiles between the two sampled groups. Because individual age and specific foraging task were not determined during collection, these differences cannot be attributed exclusively to foraging activity. Although the samples from the pear pollen-foraging group exhibited a certain degree of dispersion along the second principal component, all biological replicates clustered according to their experimental groups, further demonstrating the high reliability and biological consistency of the ribonucleic acid sequencing data.

### 3.2. Sequencing Data Quality Assessment

To further evaluate the reliability of the transcriptome sequencing data, the distribution of clean reads across different genomic regions was analyzed. As shown in Table 3, the vast majority of sequencing reads (approximately 89–94%) were successfully mapped to exonic regions, whereas only a small proportion of reads (2.2–3.6%) was mapped to intronic regions. These results indicate that most sequencing reads originated from mature messenger ribonucleic acid transcripts, demonstrating the high quality and reliability of the transcriptome sequencing data.

### 3.3. Identification of Differentially Expressed Genes

**Figure 2:**
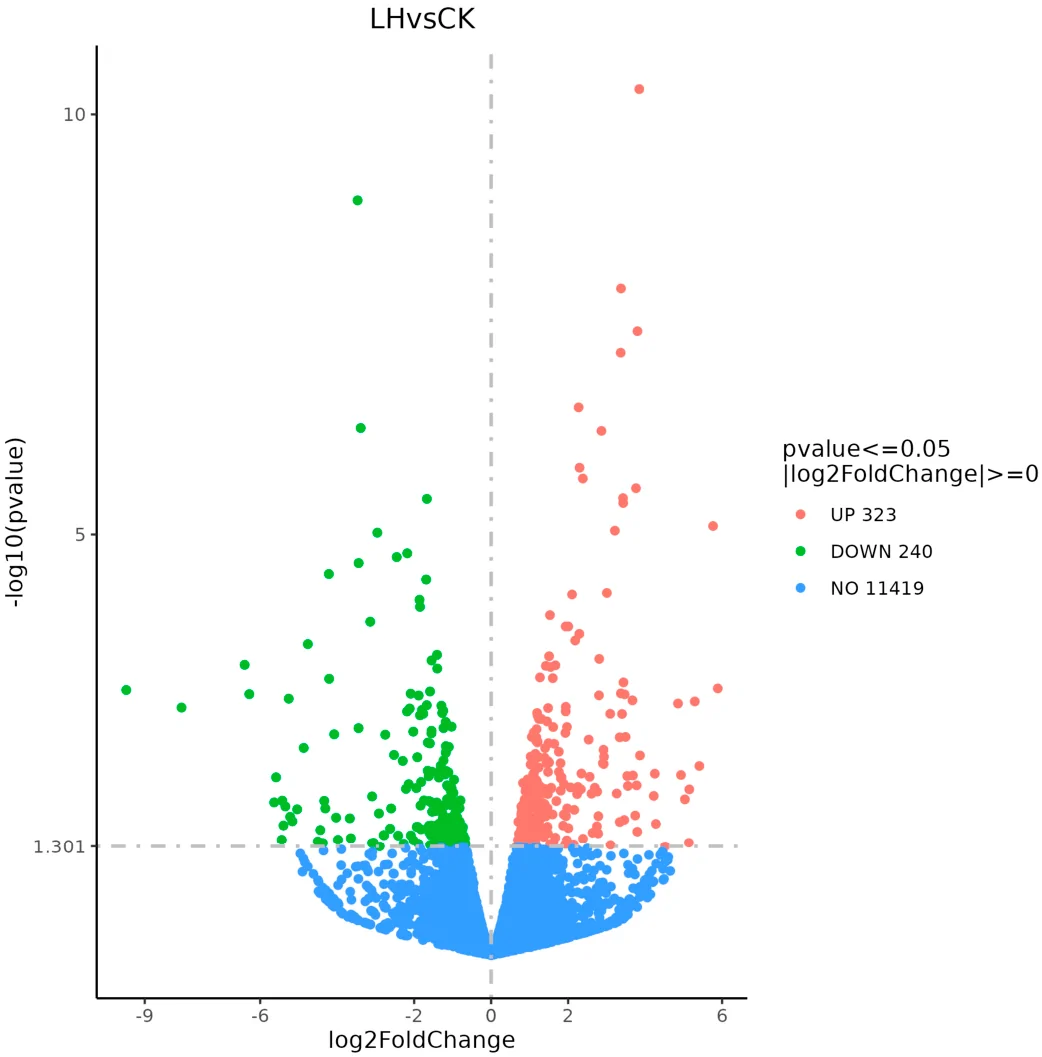
Volcano plot of differentially expressed genes in the hemolymph of *A. m. ligustica* between the pollen-foraging (LH) and non-pollen-foraging (CK) groups.

Transcriptome sequencing was performed for the two experimental groups, and differential gene expression analysis was conducted using DESeq2. As shown in Figure 2, a total of 563 differentially expressed genes were identified between the pear pollen-foraging group and the non-pollen-foraging group, including 323 upregulated genes and 240 downregulated genes.

### 3.4. Functional Classification of Differentially Expressed Genes

**Figure 3:**
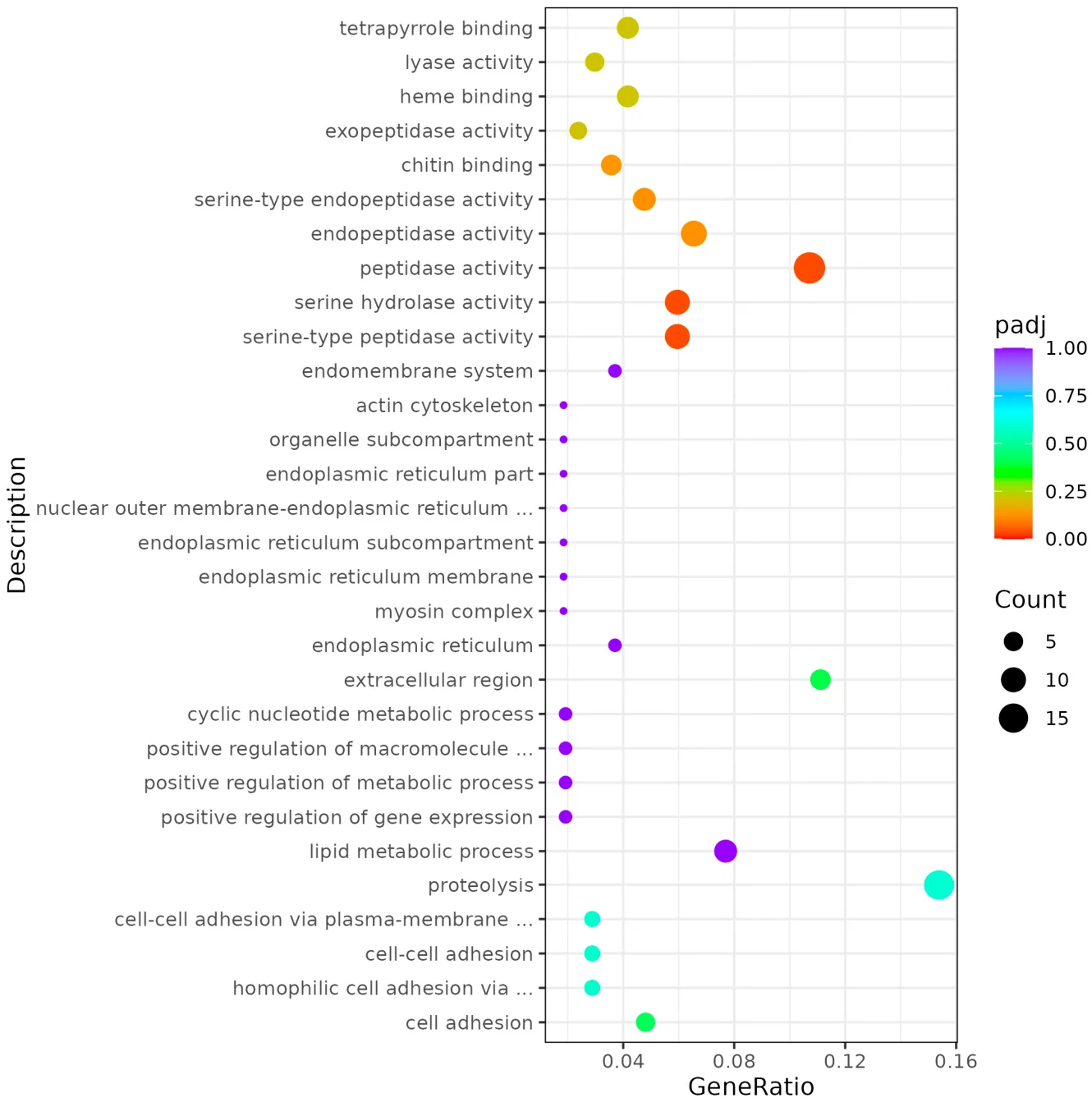
Gene Ontology enrichment analysis of differentially expressed genes in the hemolymph transcriptome of *A. m. ligustica* between the pollen-foraging (LH) and non-pollen-foraging (CK) groups.

Gene Ontology enrichment analysis was performed to characterize the functions of the differentially expressed genes identified in the hemolymph of *A. m. ligustica* from the pear pollen-foraging group and the non-foraging control group. As shown in Figure 3, the differentially expressed genes were primarily enriched in three major functional categories: peptidase/protease activity, cell adhesion, and the endomembrane system. Within the molecular function category, terms including peptidase activity, serine hydrolase activity, and endopeptidase activity were significantly enriched, exhibiting extremely high statistical significance (adjusted *p* value ≈ 0.00). Within the biological process category, proteolysis and cell–cell adhesion showed the highest GeneRatio values and contained the largest numbers of differentially expressed genes. In addition, enriched terms within the cellular component category included the endomembrane system, actin cytoskeleton, and endoplasmic reticulum, indicating that the differentially expressed genes were closely associated with intracellular membrane organization, cytoskeletal organization, and protein processing.

**Figure 4:**
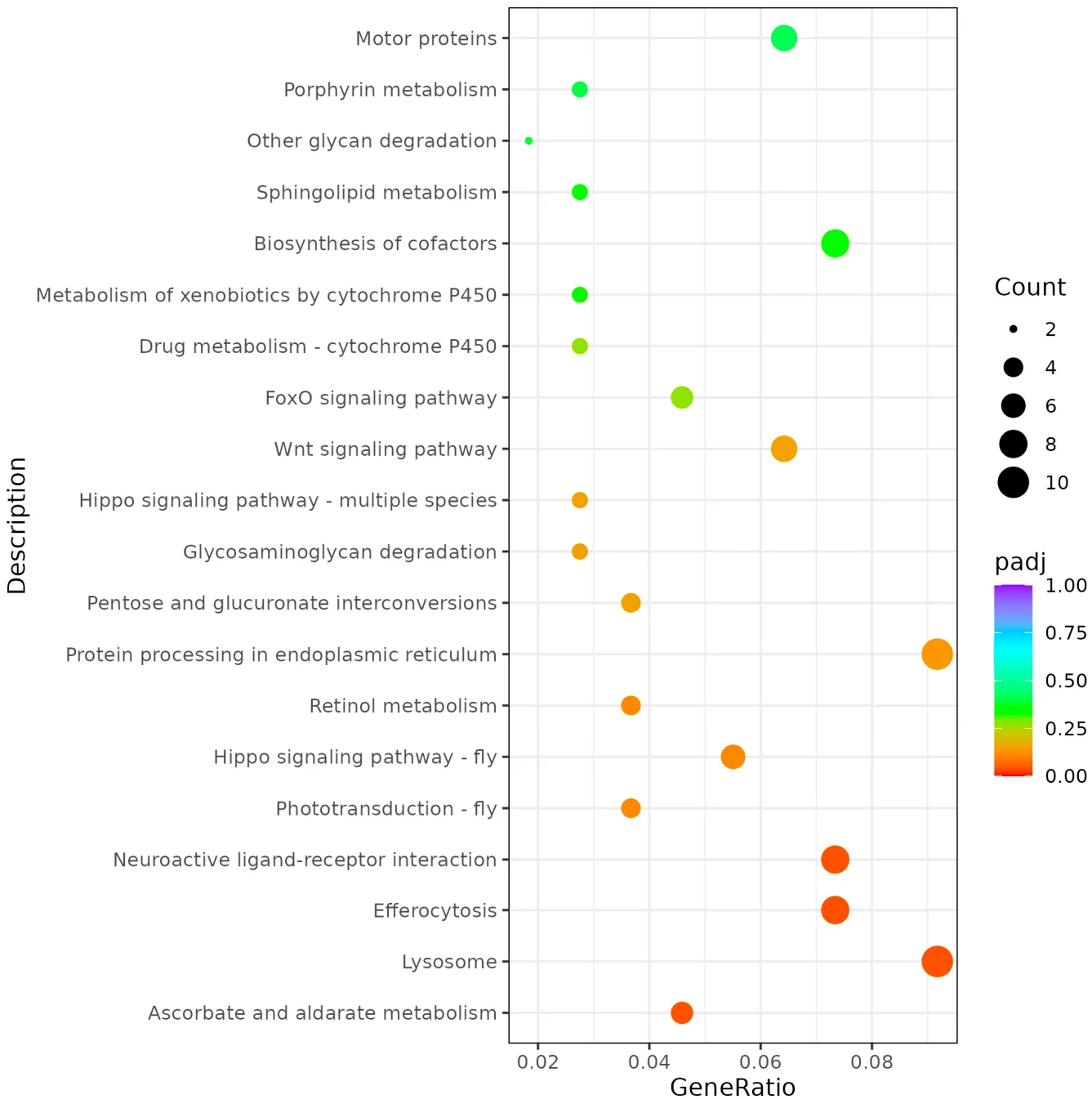
KEGG pathway enrichment analysis of differentially expressed genes in the hemolymph transcriptome of *A. m. ligustica* between the pollen-foraging (LH) and non-pollen-foraging (CK) groups.

The results of the KEGG pathway enrichment analysis are shown in Figure 4. The differentially expressed genes were primarily enriched in pathways associated with protein processing and degradation, metabolism, and signal transduction. Among these, the lysosome, efferocytosis, and protein processing in the endoplasmic reticulum pathways exhibited the highest enrichment significance and contained relatively large numbers of differentially expressed genes. This indicates that the experimental treatment markedly affected protein homeostasis and cellular clearance processes in *A. m. ligustica*.

In addition, the Forkhead box O signaling pathway, Wnt signaling pathway, and several metabolic pathways, including sphingolipid metabolism and metabolism of xenobiotics by cytochrome P450, were also significantly enriched, suggesting extensive involvement of metabolic reprogramming and signaling regulatory networks. Among the immune-related pathways, the lysosome and efferocytosis pathways contained 10 and 8 differentially expressed genes, respectively. In contrast, no significant enrichment was detected for the canonical Toll or immune deficiency signaling pathway.

### 3.5. Sequence Analysis of Immune-Related Candidate Genes in A. m. ligustica

Transcriptome analysis of the hemolymph of *A. m. ligustica* identified four immune-related candidate genes. These genes were further subjected to Basic Local Alignment Search Tool analysis (BLAST, https://blast.ncbi.nlm.nih.gov/Blast.cgi, accessed on 20 July 2026), open reading frame prediction, and transmembrane domain prediction. All four genes contained complete open reading frames. The longest open reading frame of LOC409692 was 987 base pairs in length and shared 100% sequence identity with *A. m. ligustica ADAM10-X1* (accession number: XM_026442228.1). This gene was upregulated in the pear pollen-foraging group. The longest open reading frame of LOC550695 was 798 base pairs in length and shared 100% sequence identity with *A. m. ligustica* carnitine palmitoyltransferase 1A (accession number: XM_026438847.1). This gene belongs to the acyltransferase family and was downregulated in the pear pollen-foraging group. The longest open reading frame of LOC552479 was 1001 base pairs in length and shared 100% sequence identity with *A. m. ligustica* Draper-X2 (accession number: XM_026445333.1). This gene was downregulated in the pear pollen-foraging group. The longest open reading frame of LOC411353 was 408 base pairs in length and shared 100% sequence identity with *A. m. ligustica Lipase 3* (accession number: XM_394827.6). This gene was upregulated in the pear pollen-foraging group.

### 3.6. Validation of Differentially Expressed Genes by Quantitative Real-Time Polymerase Chain Reaction (qRT-PCR)

**Figure 5:**
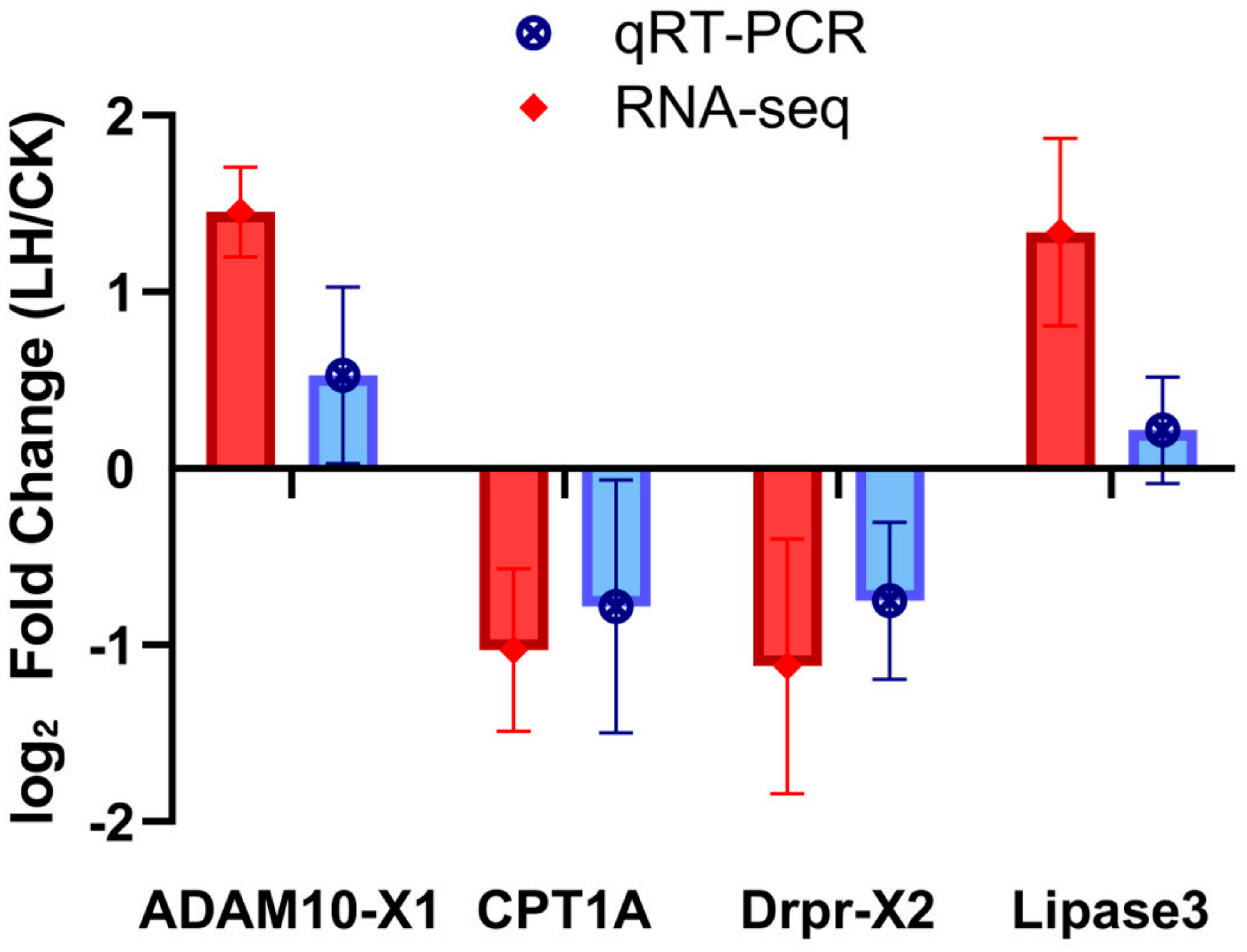
Validation of transcriptome sequencing results by *qRT-PCR* in *A. m. ligustica*. Note: Error bars represent the standard deviation of three biological replicates.

To verify the reliability of the transcriptome sequencing results, four differentially expressed genes were selected for validation using *qRT-PCR*. The expression data obtained by transcriptome sequencing and *qRT-PCR* were converted to log_2_ fold change values for the pollen-foraging group relative to the non-pollen-foraging group and are presented using a single y-axis for direct comparison (Figure 5). The results showed that the direction of differential expression (upregulation or downregulation) was completely consistent between the transcriptome sequencing and *qRT-PCR* analyses for all four genes. Minor differences in the magnitude of fold changes were attributable to the different analytical principles of the two techniques. Relatively large variations among biological replicates for some genes were likely associated with individual differences in physiological stress responses among honey bees. Overall, these findings confirmed the reliability and validity of the transcriptome sequencing results and the identification of differentially expressed genes.

## 4. Discussion

Foraging activity outside the colony is a physiologically challenging natural behavior in honey bees. During this process, worker bees not only consume substantial amounts of energy for flight but also experience a considerable input of external nutrients when returning to the colony with pollen loads [26,27]. This dual physiological pressure of “high energy consumption and high nutrient input” inevitably requires rapid responses in metabolic networks and tissue homeostasis. In the present study, by comparing the hemolymph transcriptomic differences between pollen-foraging returning workers (LH group) and returning workers without visible pollen loads (CK group), we found that the transcriptional differences between the two groups were not broadly associated with classical immune pathways (such as Toll, Imd, or the prophenoloxidase cascade), but were mainly related to metabolic and cellular homeostasis processes. Differentially expressed genes (DEGs) were significantly enriched in key metabolic and cellular renewal pathways, including lipid metabolism, proteolysis, lysosome, and efferocytosis.

PCA analysis showed that the pollen-foraging workers and returning workers without visible pollen loads were clearly separated along the PC1 direction, and biological replicates within each group exhibited good clustering, indicating the high reproducibility of the RNA-seq data. Meanwhile, these results demonstrated distinct transcriptional profiles between the two sampled groups. Further analysis identified a total of 563 differentially expressed genes, indicating substantial transcriptional differences between the two sampled groups. This result is largely consistent with previous understanding of honey bee nutritional physiology. Pollen foraging not only increases nutrient acquisition but is also accompanied by sustained flight activity, enhanced energy metabolism, and redistribution of nutritional resources among different tissues [8,28]. Therefore, as an important circulating tissue responsible for nutrient transport, material exchange, and immune defense, the molecular composition of hemolymph may undergo dynamic changes between different physiological conditions to adapt to continuously changing physiological demands [29,30,31]. Thus, the large number of differentially expressed genes observed in this study more likely reflects overall physiological differences associated with pollen-carrying status, flight activity, and other factors rather than changes in a specific signaling pathway alone. However, because the sampled workers were not age-matched and the CK group was defined by the absence of visible pollen loads, differences related to worker age or other task-related behaviors cannot be completely excluded. Notably, GO enrichment analysis in this study revealed that the differentially expressed genes were mainly associated with functional categories including proteolysis, serine hydrolase activity, and peptidase activity, rather than classical immune-related biological processes. This result indicates that the transcriptional differences between the two groups were mainly associated with fundamental metabolic processes rather than direct activation of immune responses. Protein metabolism is an essential biological process for maintaining tissue renewal, regulating enzyme activity, and facilitating nutrient utilization in insects [32]. During foraging activity, large amounts of external nutrients enter the body and subsequently require degradation, absorption, transportation, and reutilization; therefore, changes in the expression of proteases and related hydrolases have clear physiological significance [8,10].

In addition, serine proteases participate not only in digestive metabolism but also extensively regulate processes such as coagulation, melanization, and extracellular signaling, which represent an important molecular basis linking metabolism and physiological regulation [20]. Therefore, the GO analysis in this study suggests that the observed transcriptional differences may primarily manifest as changes in metabolic processes rather than broad activation of classical immune pathways.

KEGG enrichment analysis further supported the above interpretation. In this study, immune-related differentially expressed genes were mainly enriched in the lysosome and efferocytosis pathways, whereas the classical Toll and Imd signaling pathways did not show significant enrichment. Traditionally, the Toll and Imd signaling pathways are considered to primarily mediate insect defense responses against bacterial and fungal pathogens, and their activation is usually accompanied by extensive upregulation of antimicrobial peptide genes [17,33]. However, this study examined honey bees under natural field conditions without experimental pathogen infection; therefore, the absence of significant changes in classical immune pathways may reflect the physiological conditions represented by the two sampled groups. In contrast, lysosome and efferocytosis are mainly involved in intracellular degradation, organelle renewal, apoptotic cell clearance, and other fundamental biological processes, representing important components in maintaining tissue homeostasis and cellular function. Recent studies have demonstrated that lysosomes are not only intracellular degradation centers but also important regulatory platforms involved in cellular metabolism, autophagy, and immune responses [34,35]. Meanwhile, efferocytosis maintains the stability of the tissue microenvironment by efficiently removing apoptotic cells and preventing tissue damage caused by the release of cellular contents [36,37]. Therefore, the hemolymph transcriptional differences observed in this study are more likely to reflect metabolic processes and tissue homeostasis regulation associated with the physiological conditions represented by the two sampled groups rather than immune activation mediated by Toll/Imd signaling pathways under classical pathogen infection conditions.

In transcriptomic studies, screening of differentially expressed genes can only reveal molecular responses under specific physiological conditions, whereas the biological significance of candidate regulatory genes requires comprehensive interpretation based on their functional characteristics and previous studies. In this study, four candidate regulatory genes, ADAM10, Drpr, *CPT1A*, and *Lipase3*, were identified by integrating differential expression analysis, GO and KEGG enrichment results, and qRT-PCR validation. Notably, these genes were not concentrated in a single signaling pathway but were involved in multiple biological processes, including cellular renewal, protein processing, and lipid metabolism. This finding indicates that the transcriptional differences between the two sampled groups were not restricted to a single regulatory pathway but were more likely associated with multiple physiological processes.

Functional analysis of candidate genes further supported the biological characteristics revealed by GO and KEGG enrichment analyses. Although the four candidate genes participate in different molecular processes, their functions collectively point toward two core physiological processes: maintenance of tissue homeostasis and remodeling of energy metabolism. *ADAM10* is an important member of the ADAM (A Disintegrin and Metalloproteinase) family and possesses ectodomain shedding activity. Through cleavage of various cell surface receptors, adhesion molecules, and signaling proteins, *ADAM10* regulates intercellular communication, tissue renewal, and developmental processes, playing a key role in maintaining tissue homeostasis and regulating cell fate decisions [38,39]. Meanwhile, *Drpr* (Draper), a highly conserved phagocytic receptor in insects, serves as a core recognition molecule during the efferocytosis process. It mediates the recognition and clearance of apoptotic cells and damaged cellular debris by phagocytes, thereby promoting tissue repair and maintaining cellular microenvironment stability. Previous studies have demonstrated that Draper not only mediates apoptotic cell clearance but also participates in axonal clearance after neuronal injury, tissue repair, and homeostasis maintenance, suggesting that its function extends beyond traditional immune defense and is more closely involved in maintaining normal tissue homeostasis [40,41].

On the other hand, *CPT1A* and *Lipase3* are mainly involved in lipid mobilization and energy metabolism. *CPT1A* is localized on the outer mitochondrial membrane and serves as the rate-limiting enzyme responsible for the entry of long-chain fatty acids into mitochondria for β-oxidation. Its activity directly determines fatty acid oxidation efficiency and ATP production capacity, and it is therefore considered an important metabolic node regulating lipid utilization and energy supply [40,42]. *Lipase3* primarily catalyzes triglyceride hydrolysis, providing substrates for fatty acid β-oxidation and maintaining energy supply during high-energy-demand processes such as flight and foraging. Recent studies in western honey bees have further shown that lipase expression is significantly higher in the fat body of foragers than in nurse bees, indicating that behavioral transition and increased flight activity are accompanied by enhanced lipid mobilization capacity to meet the energy requirements of continuous foraging activity [43,44].

It should be noted that the candidate regulatory genes identified in this study were all derived from RNA-seq analysis, and their expression changes only reflect transcriptional differences between the two sampled groups rather than directly proving their biological functions. Therefore, this study considers ADAM10, Drpr, *CPT1A*, and *Lipase3* as candidate regulatory genes associated with the different physiological conditions represented by the two sampled groups rather than directly identifying them as key regulatory factors. Further studies combining RNA interference, tissue-specific expression analysis, protein expression detection, and enzyme activity assays are still required to verify their specific functions and regulatory mechanisms, thereby clarifying their roles in honey bee foraging behavior, physiological adaptation, and nutritional regulation. Future studies using age-matched workers and more clearly defined behavioral groups will be needed to distinguish the effects of pollen-carrying status from those associated with worker age and task-related differences. Overall, this study systematically compared the hemolymph transcriptomic profiles of pollen-foraging and non-pollen-foraging returning workers of *A. m. ligustica*, identified candidate genes with potential regulatory functions, and revealed that the transcriptional differences between the two sampled groups were mainly associated with biological processes including protein metabolism, lipid metabolism, and cellular renewal through functional annotation analysis. Unlike previous studies that have mainly focused on pathogen infection or environmental stress, this study focused on molecular responses of honey bee hemolymph under natural field conditions, providing new transcriptomic evidence for understanding physiological differences associated with pollen-carrying status and establishing a theoretical foundation for subsequent functional validation of candidate genes and studies on honey bee nutritional physiology.

## 5. Conclusions

In this study, we compared hemolymph transcriptomic profiles between pollen-foraging and non-pollen-foraging *A. m. ligustica* worker bees. Our results demonstrate that pollen-collection-related foraging activity drives broad transcriptional remodeling of hemolymph, primarily affecting metabolic homeostasis, protein degradation, lipid utilization and apoptotic-cell-clearance-related pathways. Canonical Toll and Imd immune signaling pathways were not significantly enriched under natural foraging conditions without pathogen challenge. Four candidate genes (*ADAM10-X1*, *Drpr-X2*, *CPT1A*, *Lipase3*) associated with cell homeostasis and lipid metabolism were identified and validated by qRT-PCR.

These transcriptomic findings highlight that the physiological adaptation to foraging prioritizes metabolic adjustment over classical immune-pathway activation in honey-bee hemolymph. Since our analysis is limited to transcript-level observations, further functional experiments including gene interference and protein-level detection are required to clarify the exact biological roles of these candidate genes. This work expands our understanding of honey-bee physiological plasticity under natural field foraging status and provides valuable gene resources for future honey-bee nutritional-physiology research.

## Figures and Tables

**Table 1:** Primer sequences.

Genes	Forward Primer Sequences	Reverse Primer Sequences
*ADAM10-X1*	GGGAAGAAGCGCGAATCAAC	AACGGGATCCCTCCGTCTAA
*CPT1A*	GCAGAACATTCATGTTACAAAGAAG	CCATTGGAGGCGTACAGGAG
*Drpr-X2*	TGCGCCAGGATATAGAGGTG	ATGTGCATGTGCCTGCTGTA
*Lipase3*	TGCGTAGTGCCGTTGATTTTG	CCTCCTCGCGTGCTAATTCT
Arp1	TGCTGCACTCGTAGTTGACAATGG	ACCCTGGTGGCGTGGTCTTC

**Table 2:** Quality assessment statistics of the hemolymph transcriptome sequencing data from *A. m. ligustica.* Note: LH1–LH3 represent the three biological replicates of the pollen-foraging group, whereas CK1–CK3 represent the three biological replicates of returning workers without visible pollen loads.

Sample	Raw Reads	Raw Bases	Clean Reads	Clean Bases	Error Rate	Q20%	Q30%	GCpct
LH1	59,433,840	8.92 G	57,811,344	8.67 G	0.01	99.39	97.91	36.32
LH2	48,428,246	7.26 G	47,146,436	7.07 G	0.01	99.4	97.93	37.49
LH3	48,706,274	7.31 G	47,102,406	7.07 G	0.01	99.42	97.95	37.38
CK1	58,241,750	8.74 G	55,649,332	8.35 G	0.01	99.39	97.91	35.57
CK2	49,212,538	7.38 G	47,554,422	7.13 G	0.01	99.57	98.32	37.95
CK3	50,449,242	7.57 G	48,578,420	7.29 G	0.01	99.57	98.34	37.46

**Table 3:** Summary of the alignment of hemolymph transcriptome sequencing reads from *A. m. ligustica* to the reference genome.

Sample	Exon	Intron	Intergenic
LH1	6,969,382,072 (90.0281%)	225,187,671 (2.9089%)	546,769,421 (7.0630%)
LH2	5,774,376,561 (91.1287%)	167,899,679 (2.6497%)	394,230,372 (6.2216%)
LH3	5,641,919,748 (90.6278%)	170,308,936 (2.7357%)	413,149,602 (6.6365%)
CK1	6,160,018,015 (89.3150%)	251,070,436 (3.6403%)	485,869,625 (7.0447%)
CK2	5,103,591,997 (93.0312%)	124,425,121 (2.2681%)	257,877,851 (4.7007%)
CK3	6,085,679,447 (94.6207%)	141,502,454 (2.2001%)	204,480,824 (3.1793%)

## Data Availability

The RNA-seq datasets generated and analyzed during the current study have been deposited in the NCBI Sequence Read Archive (SRA) database under accession number PRJNA1505795. The datasets are currently under embargo. All other data supporting the findings of this study are available from the corresponding author upon reasonable request.

## References

[1] Ollerton J., Winfree R., Tarrant S. (2011). How many flowering plants are pollinated by animals?. Oikos.

[2] Potts S.G., Imperatriz-Fonseca V., Ngo H.T., Aizen M.A., Biesmeijer J.C., Breeze T.D., Dicks L.V., Garibaldi L.A., Hill R., Settele J. (2016). Safeguarding pollinators and their values to human well-being. Nature.

[3] Klein A.M., Vaissiere B.E., Cane J.H., Steffan-Dewenter I., Cunningham S.A., Kremen C., Tscharntke T. (2007). Importance of pollinators in changing landscapes for world crops. Proc. Biol. Sci..

[4] Vanengelsdorp D., Evans J.D., Saegerman C., Mullin C., Haubruge E., Nguyen B.K., Frazier M., Frazier J., Cox-Foster D., Chen Y. (2009). Colony Collapse Disorder: A Descriptive Study. PLoS ONE.

[5] Neumann P., Carreck N.L. (2010). Honey bee colony losses. J. Apic. Res..

[6] Goulson D., Nicholls E., Botias C., Rotheray E.L. (2015). Bee declines driven by combined stress from parasites, pesticides, and lack of flowers. Science.

[7] Benjamin D., Evans J.D., Ping C.Y., Laurent G., Peter N., Aguilar P.V. (2012). Predictive Markers of Honey Bee Colony Collapse. PLoS ONE.

[8] Brodschneider R., Crailsheim K. (2010). Nutrition and health in honey bees. Apidologie.

[9] Garance D.P., Marion S., Yves L.C., Belzunces L.P., Axel D., André K., Séverine S., Jean-Luc B., Cédric A., Jochen Z. (2013). Influence of Pollen Nutrition on Honey Bee Health: Do Pollen Quality and Diversity Matter?. PLoS ONE.

[10] Wright G.A., Nicolson S.W., Shafir S. (2017). Nutritional Physiology and Ecology of Honey Bees. Annu. Rev. Entomol..

[11] Alaux C., Ducloz F., Crauser D., Conte Y.L. (2010). Diet effects on honeybee immunocompetence. Biol. Lett..

[12] Bry M.S., Olszewski K., Bartoń M., Strachecka A.J.A. (2025). Changes in the Activities of Antioxidant Enzymes in the Fat Body and Hemolymph of *Apis mellifera* L. Due to Pollen Monodiets. Antioxidants.

[13] Bry M.S., Staniec B., Strachecka A.J.S.R. (2024). The effect of pollen monodiets on fat body morphology parameters and energy substrate levels in the fat body and hemolymph of *Apis mellifera* L. workers. Sci. Rep..

[14] Bryś M.S., Olszewski K., Strachecka A. (2025). The relationship between pollen monodiets and the activities of proteolytic systems in the fat body and hemolymph of honeybee workers. PLoS ONE.

[15] Chapman R.F.C. (1971). The Insects: Structure and Function.

[16] Hillyer J.F. (2016). Insect immunology and hematopoiesis. Dev. Comp. Immunol..

[17] Lemaitre B., Hoffmann J. (2007). The Host Defense of *Drosophila melanogaster*. Annu. Rev. Immunol..

[18] Evans J.D., Aronstein K., Chen Y.P., Hetru C., Imler J., Jiang H., Kanost M., Thompson G.J., Zou Z., Hultmark D. (2010). Immune pathways and defence mechanisms in honey bees *Apis mellifera*. Insect Mol. Biol..

[19] Lavine M.D., Strand M.R. (2002). Insect hemocytes and their role in immunity. Insect Biochem. Mol. Biol..

[20] Kanost M.R., Jiang H. (2015). Clip-domain serine proteases as immune factors in insect hemolymph. Curr. Opin. Insect.

[21] Jiang H., Kanost M.R. (2000). The clip-domain family of serine proteases in arthropods. Insect Biochem. Mol. Biol..

[22] Skaar E.P., Ruiz V. (2020). Iron sequestration by transferrin 1 mediates nutritional immunity in *Drosophila melanogaster*. Proc. Natl. Acad. Sci. USA.

[23] Margotta J.W., Mancinelli G.E., Benito A.A., Ammons A., Roberts S.P., Elekonich M.M. (2012). Effects of Flight on Gene Expression and Aging in the Honey Bee Brain and Flight Muscle. Insects.

[24] Margotta J.W., Roberts S.P., Elekonich M.M. (2018). Effects of flight activity and age on oxidative damage in the honey bee, *Apis mellifera*. J. Exp. Biol..

[25] Schulz D.J., Tanya P., Kim F.M., Robinson G.E., Page R.E. (2004). Comparisons of Juvenile Hormone Hemolymph and Octopamine Brain Titers in Honey Bees (Hymenoptera: Apidae) Selected for High and Low Pollen Hoarding. Ann. Entomol. Soc. Am..

[26] Li X., Zhang J.Y., Gao W.Y., Wang Y., Huang L.Q. (2012). Chemical Composition and Anti-inflammatory and Antioxidant Activities of Eight Pear Cultivars. J. Agric. Food Chem..

[27] Vaudo A.D., Tooker J.F., Grozinger C.M., Patch H.M. (2015). Bee nutrition and floral resource restoration. Curr. Opin. Insect Sci..

[28] Vaudo A.D., Tooker J.F., Patch H.M., Biddinger D.J., Grozinger C.M. (2020). Pollen Protein: Lipid Macronutrient Ratios May Guide Broad Patterns of Bee Species Floral Preferences. Insects.

[29] Alaux C., Dantec C., Parrinello H., Conte Y.L. (2011). Nutrigenomics in honey bees: Digital gene expression analysis of pollen’s nutritive effects on healthy and varroa-parasitized bees. BMC Genom..

[30] Harrison J.F., Roberts S.P. (2000). Flight respiration and energetics. Annu. Rev. Physiol..

[31] Feng M., Ramadan H., Han B., Fang Y., Li J. (2014). Hemolymph proteome changes during worker brood development match the biological divergences between western honey bees (*Apis mellifera*) and eastern honey bees (*Apis cerana*). BMC Genom..

[32] Elfar S.A., Bahgat I.M., Shebl M., Lihoreau M., Tawfik M.M. (2023). Intraspecific proteomic profiling and potential biological activities of the honey bee hemolymph. bioRxiv.

[33] Bogaerts A., Baggerman G., Vierstraete E., Schoofs L., Verleyen P. (2009). The hemolymph proteome of the honeybee: Gel-based or gel-free?. Proteomics.

[34] Nation J.L. (2008). Insect Physiology and Biochemistry.

[35] Myllymaki H., Valanne S., Ramet M. (2014). The Drosophila Imd Signaling Pathway. J. Immunol..

[36] Ballabio A., Bonifacino J.S. (2019). Lysosomes as dynamic regulators of cell and organismal homeostasis. Nat. Rev. Mol. Cell Biol..

[37] Lawrence R.E., Zoncu R. (2019). The lysosome as a cellular centre for signalling, metabolism and quality control. Nat. Cell Biol..

[38] Elliott M.R., Ravichandran K.S. (2016). The Dynamics of Apoptotic Cell Clearance. Dev. Cell.

[39] Boada-Romero E., Martinez J., Heckmann B.L., Green D.R. (2020). The clearance of dead cells by efferocytosis. Nat. Rev. Mol. Cell Biol..

[40] Weber S., Saftig P. (2012). Ectodomain shedding and ADAMs in development. Development.

[41] Reiss K., Saftig P. (2009). The “A Disintegrin and Metalloprotease” (ADAM) family of sheddases: Physiological and cellular functions. Semin. Cell Dev. Biol..

[42] Logan M.A., Freeman M.R. (2007). The scoop on the fly brain: Glial engulfment functions in Drosophila. Neuron Glia Biol..

[43] Yeon Ryu S., Hui Kim Y., Myung Kim J., Yeon Kim B., Sik Lee K., Rae Jin B. (2022). Molecular cloning and characterization of a lipase from the honeybee *Apis mellifera*. J. Asia-Pac. Entomol..

[44] Macdonald J.M., Beach M.G., Porpiglia E., Sheehan A.E., Freeman M.R. (2006). The Drosophila Cell Corpse Engulfment Receptor Draper Mediates Glial Clearance of Severed Axons. Neuron.

